# Prognostic factors for surgical outcome in spinal cord injury associated with ossification of the posterior longitudinal ligament (OPLL)

**DOI:** 10.1186/s13018-015-0235-3

**Published:** 2015-06-12

**Authors:** Soon Young Kwon, Jun Jae Shin, Ji Hae Lee, Woo Ho Cho

**Affiliations:** Department of Neurosurgery, Sanggye Paik Hospital, Inje University College of Medicine, Sanggye-7 dong, 761-1, Nowon-gu, Seoul 139-707 South Korea; Department of Radiology, Sanggye Paik Hospital, Inje University College of Medicine, Sanggye-7 dong, 761-1, Nowon-gu, Seoul 139-707 South Korea

**Keywords:** Ossification of the posterior longitudinal ligament, Spinal cord injury, Magnetic resonance imaging

## Abstract

**Background:**

Ossification of the posterior longitudinal ligament (OPLL) may increase the risk of spinal cord injury (SCI) with various neurological deficits after minor trauma. However, few studies have investigated the influence of OPLL on neurological outcome after acute cord injury. We examined whether severe spinal canal stenosis caused by OPLL affects neurological outcome after SCI based on intramedullary signal intensity (SI) changes on magnetic resonance imaging (MRI).

**Methods:**

From June 2006 to July 2013, we treated 246 patients with cervical cord injury. Fifty-one (20.7 %) patients had ventral cord compression due to OPLL without any bony fractures. Among them, 38 patients (34 men, mean age 62.7 years) underwent cervical laminoplasty (8) and cervical decompression and fixation (30). The neurologic assessments were performed in patients who had 1-year follow-up, and the mean follow-up period was 42.2 months. OPLL type, cause of injury, cervical sagittal angle, cervical spine stenosis, cord compression ratio (space available for the spinal cord (SAC)), and grade of intramedullary SI (grade 0, none; grade 1, light; grade 2, intense T2WI) were assessed.

**Results:**

Mean American Spinal Injury Association (ASIA) motor score at admission was 38.4 ± 21.9 (range, 2–70) and improved to 67.7 ± 19.1 (range, 8–94) at last follow-up (*p* < 0.05). Mean recovery rate of the motor score was 55.8 ± 19.9 %. Five patients had SI grade 0, 20 patients had SI grade 1, and 13 patients had SI grade 2. Among the variables tested, age, initial ASIA motor grade, intramedullary SI grade, and SAC were significantly related to neurological outcome. However, initial cervical alignment, canal diameter, length of SI, time interval between injury and operation, and OPLL type had no significant effect on neurological outcome.

**Conclusions:**

Preoperative neurological status, cord compression ratio, and SI grade are related to neurological outcome in patients with SCI associated with OPLL. The better the preoperative neurological status, the more favorable the neurological outcome after surgery. A higher SI grade on preoperative T2WI was negatively related to neurological outcome. Therefore, the severity of SI change, cord compression ratio, and preoperative neurological status can be regarded as significant prognostic factors in patients with SCI associated with OPLL.

## Introduction

Ossification of the posterior longitudinal ligament (OPLL) is a common cervical disease that can cause stenotic changes in the spinal canal and contribute to the development of quadriparesis. OPLL has been found in about 30 % of patients with spinal cord injury (SCI) [1, 2]. These results indicate that OPLL is a major risk factor for developing acute cervical SCI. Several factors for predicting surgical outcome after surgical treatment of cervical OPLL have been discussed [3–6]. Some authors reported that patients with increased intramedullary signal intensity (SI) based on magnetic resonance imaging (MRI) had a poor prognosis after surgical decompression, while others found no clear relationship between SI and prognosis after surgery [3, 5–9]. Only a handful of studies have investigated prognostic factors for SCI associated with OPLL [2, 10, 11]. It is difficult to predict the recovery rate and prognosis of SCI with OPLL because traumatic cervical SCI patients are a heterogeneous patient population and are treated with various management methods.

We performed a retrospective investigation to examine prognostic factors in SCI associated with OPLL without any bony fracture. We assessed whether severe spinal canal stenosis caused by OPLL, intramedullary signal change on magnetic resonance images, or other potential prognostic factors affected neurological outcome after SCI without any bony fractures.

## Materials and methods

### Patient selection

This study was approved by the local ethical committee and the institutional review board of our hospital. From June 2006 to July 2013, we treated 246 patients with cervical cord injury. Among the patients treated, 51 patients (20.7 %) had ventral cord compression involving more than three-level OPLL without bony fractures. Thirteen patients who underwent anterior cervical decompression were excluded because of bias or differences in surgical factors.

Thirty-eight patients (34 men) with a mean age of 62.7 years (range, 40 to 82 years) had spinal cord injury without any bony fractures associated with more than three-level OPLL. All patients underwent posterior cervical decompressive surgery using cervical laminoplasty or laminectomy with fusion.

Patient age, gender, type of trauma, duration of symptoms, neurologic deficits, medical conditions, and radiographic findings were investigated. Charts were reviewed to determine the cause of injury and the interval between the accident and operation. Force was confirmed by edema of prevertebral soft tissue on MRI or frontal ecchymosis. Presence of OPLL was confirmed by X-ray or computed tomography.

Inclusion criteria were cervical cord injury and admission through the outpatient department (OPD) or emergency room (ER). Patients with spinal cord compression caused by OPLL extending more than three vertebrae were included, as were those with the specific incomplete neurological syndrome called central cord syndrome, which is characterized by greater muscle weakness and/or sensory loss in the upper limbs than the lower limbs and others caused by hyperextension, hyperflexion, or axial loading injuries.

We excluded patients with neurological symptoms before trauma, one- or two-level OPLL, other acute traumatic cord injuries with cervical bony fracture or dislocation caused by high-energy trauma, complete neurological injury after trauma, or another confirmed neurological disorder (e.g., cerebral palsy, Parkinson’s disease, multiple sclerosis, polio). Ossification of the yellow ligament, severe disc herniation, and previous history of cervical spine surgery were also exclusion criteria.

All patients underwent high-resolution MR imaging using a 1.5 T Signa (Siemens Medical System) imaging unit prior to surgery. We carefully ruled out tumors, demyelinating disease, sarcoid lesions, and spinal cord infarct. Patients who died or had missing data before follow-up evaluation were excluded.

We defined complications as events that occurred within 1 month after surgery. We analyzed neurologic recovery in patients who had completed at least 12 months of follow-up.

### Neurological evaluation

Neurological status was assessed using the International Standards for Neurological and Functional Classification of Spinal Cord Injury according to the American Spinal Injury Association (ASIA). ASIA impairment scale and motor score (ranging from 0 to 100) were documented at the time of admission and discharge for each patient, and at about 12 months after surgery. The neurological assessments were performed in patients who had completed 1-year follow-up (5 patients underwent 12-month follow-up postoperatively and 33 patients 24-month follow-up after surgery).

Improvement in neurologic score was determined by calculating the recovery rate using the method of Lucas and Ducker [12]. Recovery rate (RR) takes into account the impact of changes dependent on baseline values by implying the largest potential improvement: RR = (NS_f_ − NS_i_) / (*T* − NS_i_), where RR is the recovery rate, NS_f_ is the neurologic score at follow-up, NS_i_ is the initial neurologic score, and *T* is the maximum possible score (100, 114, and 114 for the motor score, sensory score, and pin-prick score, respectively).

### Radiological evaluation

Preoperative plain radiographs, three-dimensional computed tomography (3D CT), and MRI of the cervical spine were performed in all patients. Radiographic parameters include the type of OPLL, sagittal diameter of the canal, cervical lordosis, space available for the spinal cord (SAC), and intramedullary signal intensity on T2-weighted MR images were recorded. OPLL was classified as segmental type, continuous type, mixed type, or localized type according to the criteria of the Japanese Investigation Committee on the Ossification of the Spinal Ligament [13].

**Fig. 1:**
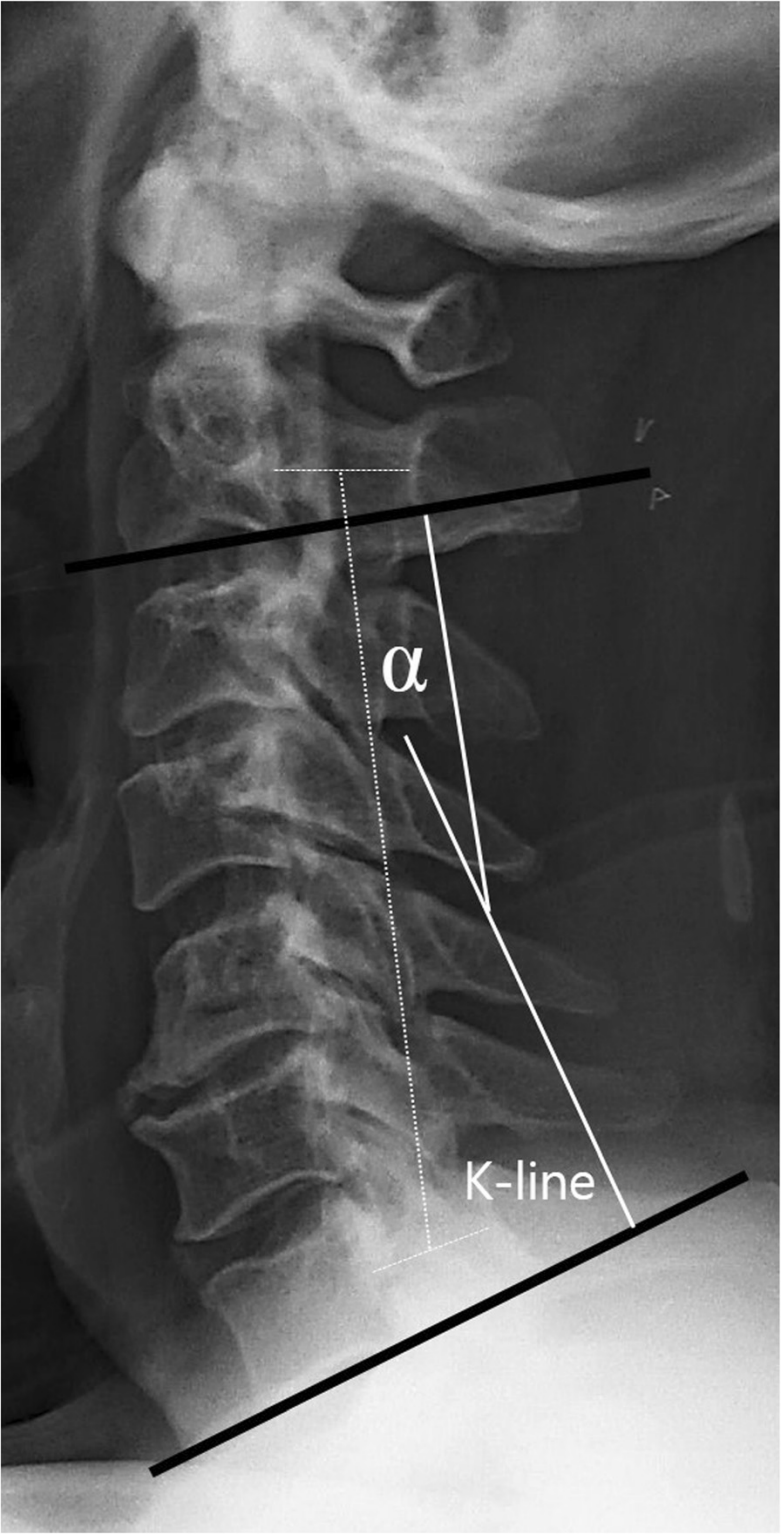
Cervical lordosis and K-line. Lordotic angle was created by drawing a *line parallel to the inferior aspect of the C2 body* and a *line parallel to that of the C7 body* on lateral view with the patient in a neutral position. The *K-line* is a straight line from the midpoints of the spinal canal at C2 and C7

Cervical spine stenosis was measured by the Torg-Pavlov ratio (ratio of the sagittal spinal canal diameter to the vertebral body diameter at the same level) on plain radiographs. Cervical sagittal alignment (lordotic angle) was determined according to Cobb’s method by drawing a line parallel to the inferior aspect of the C2 body and a line parallel to that of the C7 body from a neutral lateral view. Also, the K-line slope [14], the straight line of the midpoints from C2 to C7 spinal canal, was used as a radiological parameter (Fig. 1).

**Fig. 2:**
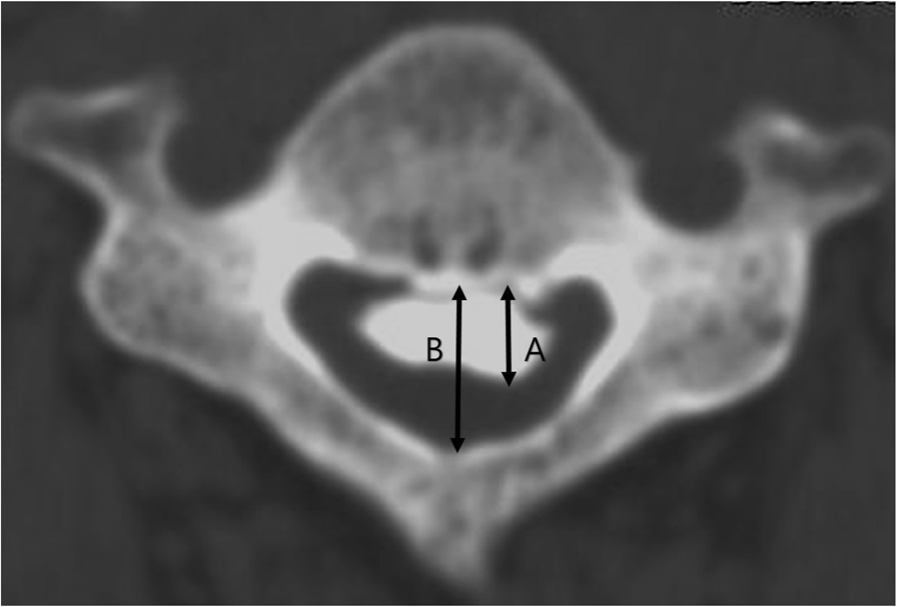
Measurement of the space available for the spinal cord (SAC). SAC was calculated on the basis of axial computed tomography. *B* = developmental canal diameter. *B-A* = space available for the spinal cord (SAC)

A computed tomographic (CT) scan was performed before surgery for all patients. An Aquilion 16 CT scanner (Toshiba Medical Systems, Tokyo, Japan) was used in this series. Horizontal slices were cut to a slice thickness of 1 mm in all cases. Three-dimensional CT was performed at a neutral cervical position to avoid neurological deterioration in cervical injured patients with cervical OPLL. Using axial CT scans, we evaluated static canal stenosis caused by OPLL by measuring the SAC (Fig. 2).

**Fig. 3:**
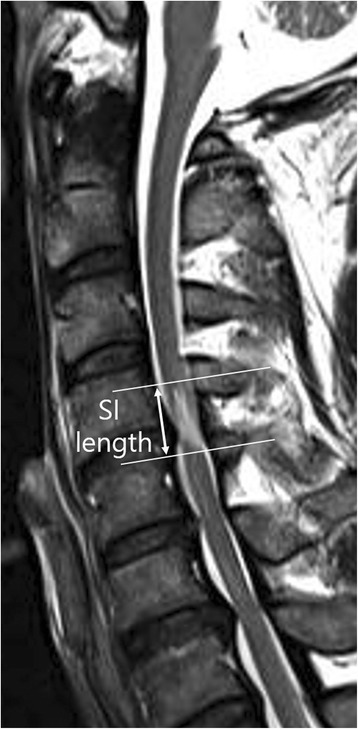
Length of signal intensity. It was measured by the proximal-distal range of the intramedullary hyperintensity on T2-weighted images

Using a picture archiving and communication system (PACS), we measured the length of the signal change from one end of the involved region to the other on T2-weighted sagittal images (Fig. 3).

**Fig. 4:**
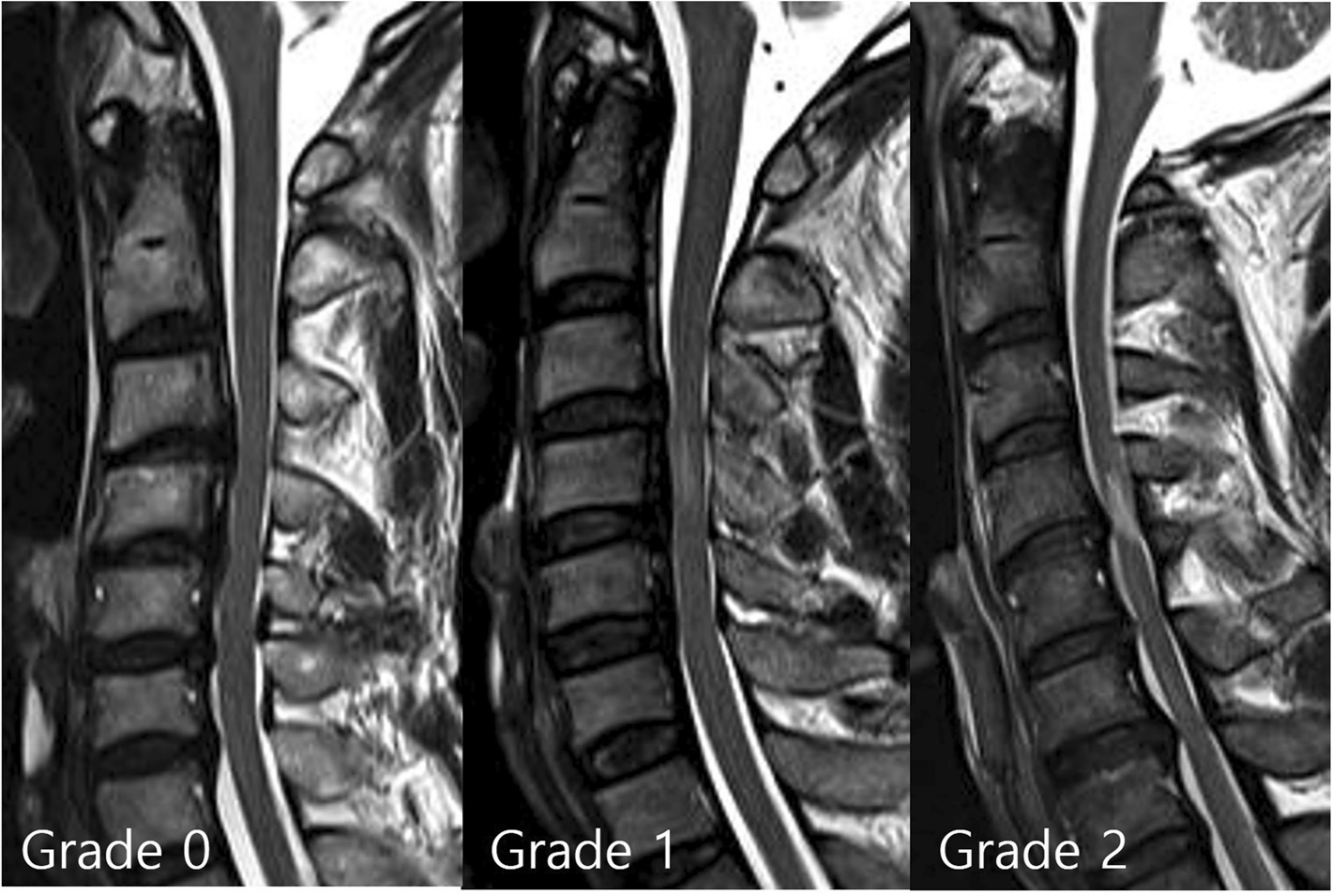
Signal intensity grade. Signal intensity of the spinal cord was assessed as grade 0—no change in signal intensity (SI) on T2-weighted MR images; grade 1—light intensity change; and grade 2—bright signal, clearly distinguishable from that of grade 1

We divided the signal intensity (SI) on T2-weighted MR images into three grades. Increased SI of the spinal cord at the narrowest level was assigned one of three grades (grades 0, 1, or 2) by experienced radiologists. Observers were blinded to the patient data. SI was classified as grade 0 if there was no intramedullary high SI on T2-weighted MR images, grade 1 if a predominantly faint and fuzzy border was noted, and grade 2 if a predominantly intense and well-defined border was noted (Fig. 4).

### Surgical management

We attempted to perform cervical decompressive surgeries in patients with neurological deficits as soon as possible. If the spinal cord was injured within 8 h, patients with neurological deficit after minor trauma received a high dose of prednisolone, according to the American National Acute Spinal Cord Injury Society criteria [15]. The first dose was 30 mg/kg and given within 15 min; after 45 min, a second dose of 5.4 mg (kg/h) was maintained for 23 h. If the injury time exceeded 8 h, 20 mg dexamethasone was used four times a day for the first 3 days and daily reduced for the next 3 days. The decision to perform decompressive surgery was based on the patient’s general and neurological status. We defined the operation time as the time interval between the traumatic event and surgery.

Cervical extensive laminoplasty was selected based on the neurologic symptoms and the extent of cord compressed levels, including the most narrowed side on images. Unilateral or bilateral gutters were made using a high-speed drill at the area between the facet joints and the laminae, and spinous processes were split sagittally with a high-speed burr. The spinal canal was enlarged by opening the split laminae unilaterally or bilaterally with a spreader. Posterior cervical titanium miniplates (Centerpiece®, Medtronic Sofamor Danek, Memphis, TN, USA) or hydroxyapatite spacers (Apacerum®, Asahi Optical Co., Ltd., Tokyo, Japan) were used to keep the “door” open.

For OPLL patients with local kyphosis or segmental instability, we performed cervical laminectomy and fusion and tried to realign local kyphosis using a posterior screw-rod system. Following complete decompression of neural elements, lateral mass screw and rod instrumentation were performed. Perizygapophysial joints were filled with autologous bone fragments from the excised lamina. After surgery, all patients were required to wear Miami neck collars or Philadelphia braces for a mean period of 3 months. Patients were followed clinically by radiography using plain and dynamic films at 1, 3, and 6 months and then every 6 months postoperatively.

### Statistical analysis

Multifactorial effects of variables were studied. Mann-Whitney *U* tests were used for nonparametric analyses of differences between pairs of groups, and the Kruskal-Wallis test followed by the Mann-Whitney *U* test was used to examine differences between the three SI groups. To identify variables independently related to ASIA recovery ratio, univariate or multivariate logistic regression analyses were used. Pearson’s correlation analysis was performed to determine the significance of the correlation between ASIA recovery ratio and age, duration of symptoms, cervical alignment, cervical canal stenosis (Pavlov’s ratio), SAC, preoperative ASIA motor score, SI length, and postoperative ASIA motor score. Spearman’s correlation analysis was performed to determine the significance of the correlation between ASIA recovery ratio and gender, OPLL type, or preoperative SI grade. Correlations between variables were assessed with regression analyses using MedCalc version 14.0 (MedCalc, Mariakerke, Belgium). *p* values < 0.05 were considered significant.

## Results

The primary cause of cord injury in patients with OPLL was slipping down and falling injuries (78.9 %), followed by a motor vehicle accident. The patient had episodes of minor neck trauma: injuries from slipping down in 23, falling down in 7 patients, motor vehicle accident in 7, being struck by objects in 1, and sports activities in 2. The injury was mostly caused by a sudden force with subsequent compression of the cord by a bony posterior ligament or narrowed canal. Most cases of the cervical SCI complicated by OPLL are caused by hyperextension injuries resulting from falling or slipping down injuries. Fourteen patients had typical central cord syndrome. Four had incomplete cord injuries caused by flexion injuries, and 20 had incomplete neurological deficits by combined injuries such as lateral bending, rotation, axial loading, and hyperflexion or hyperextension of the cord. All 38 patients included in this study underwent posterior decompressive surgery. Expansive cervical laminoplasty was performed in 8 patients, and cervical laminectomy with fusion was performed in 30 patients.

Mean ASIA motor score at admission was 38.4 ± 21.9 (range, 2–70) and improved to 67.7 ± 19.1 at last follow-up (range, 8–94) (*p* < 0.05). Overall, the mean recovery rate of the motor score was 55.8 ± 19.9 % (range, 8.2–92.8 %), and the recovery ratio improved in all patients. In addition, the average ASIA score of light touch sensation and pin-prick sensation increased from 70.9 ± 34.5 and 70.9 ± 34.4, respectively, at admission to 79.7 ± 34.1 and 79.7 ± 34.1, respectively, after the final follow-up. Mean recovery rate of light touch and pin sensation were 32.3 ± 28.2 % and 33.8 ± 24.7 %, respectively. In general, the sensory recovery ratio was lower than the motor recovery ratio. Mean follow-up time was 42.2 ± 25.1 months (range, 16.8–97.8 months). The duration of preoperative symptoms ranged from 1 to 23 days. SAC was 6.1 ± 2.2 mm (range, 2.6–10.6 mm). The mean length of SI changes was 5.9 ± 3.7 mm (range, 0–13.9 mm).

Radiological findings revealed continuous in 14 patients, mixed type OPLL in 4 patients, and segmental type OPLL in 20 patients. There was no significant relationship between OPLL type and neurological outcome in our study. The mean OPLL level treated by surgical operation was 3.2 ± 0.4. Among 38 patients, 33 (86.8 %) showed increased signal intensity within the spinal cord on T2-weighted MR images, whereas 5 patients (13.2 %) did not. SI change on MR images was located on C3/4 level in 13 patients, C4/5 in 14, C5/6 in 4, C2/3/4 in 1 and C3/4/5 in 1. The mean length of SI changes was 5.9 ± 3.7 mm (range, 0-13.9 mm). However, the length of pathological changes on spinal cord was not correlated with neurological outcome. When the grade of intramedullary SI change on T2WI was assessed, 5 patients showed SI grade 0 changes, 20 patients SI grade 1 changes, and 13 patients grade 2 changes. In this study, there was no low-signal change on T1-weighted image initially.

The respective surgical level caused by OPLL was 3.4 ± 0.5 in grade 0 patients, 3.1 ± 0.3 in grade 1, and 3.2 ± 0.4 in grade 2. There was no significant difference in surgical levels among the three groups. The surgical procedures performed were expansive laminoplasty in one patient with grade 0 and cervical laminectomy with fusion in four patients with grade 0. In grade 1, expansive laminoplasty was performed in 4 patients and cervical laminectomy with fusion in 16. In grade 2, expansive laminoplasty was performed in three patients and cervical laminectomy with fusion in ten (Table 1).

**Table 1:** Clinical characteristics and neurological outcome according to the severity of signal intensity

	Grade 0 (*n* = 5)	Grade 1 (*n* = 20)	Grade 2 (*n* = 13)	*p* value
Age (years)	57.4 ± 6.1	63.8 ± 11.5	63.2 ± 11.4	0.523 NS
Symptom duration (days)	3.8 ± 4.4	3.8 ± 4.9	2.2 ± 1.7	0.634 NS
SAC (mm)	7.2 ± 1.5	6.6 ± 2.4	4.9 ± 1.6	0.056 NS
Cervical curvature (°)	13.3 ± 4.1	15.1 ± 6.9	16.8 ± 9.8	0.769 NS
Cervical canal stenosis	0.84 ± 0.27	0.69 ± 0.17	0.74 ± 0.18	0.174 NS
Preoperative motor score	60.0 ± 10.1	40.6 ± 21.1	26.0 ± 6.9	0.033
Preoperative touch score	100.8 ± 7.6	77.5 ± 29.2	49.4 ± 36.7	0.003
Recovery motor rate (%)	82.4 ± 18.1	66.0 ± 18.1	30.9 ± 26.6	0.0005
Recovery touch rate (%)	69.8 ± 19.7	29.7 ± 19.2	21.8 ± 22.2	0.0009
Surgical levels	3.4 ± 0.5	3.1 ± 0.3	3.2 ± 0.4	0.267 NS
Laminectomy and fusion	4	16	10	
Expansive laminoplasty	1	4	3	

*SAC* space available for the spinal cord, *NS* not significant

**Fig. 5:**
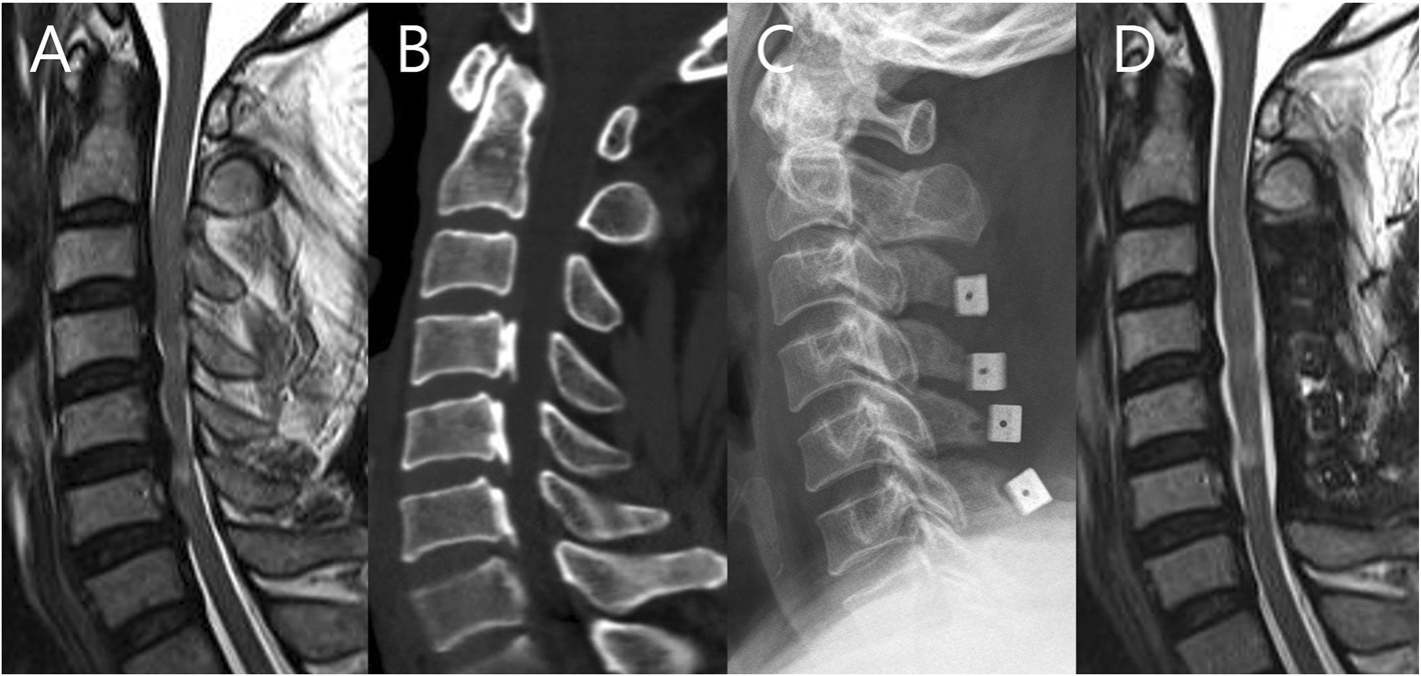
A patient with intramedullary signal intensity grade 2. **a** Preoperative T2-weighted MRI results for a 54-year-old male patient with spinal cord compression at C3-4-5-6-7. Intramedullary high signal intensity (SI), grade 2, was visible at the region of the marked compressed cord. Initial ASIA motor score was 48. **b** Cervical 3D CT showed mixed type ossification of the posterior longitudinal ligament (OPLL). Spinal canal stenosis (SAC) was 5.9 mm. **c** The patient underwent cervical laminoplasty at C3-4-5-6. **d** MRI of the patient from B 12 months after cervical laminoplasty with successful decompression of the spinal cord. The patient’s ASIA motor score was 72. The recovery rate was 46.2 %

Preoperative motor scores and recovery ratios (%) were 60.0 ± 10.1 and 82.4 ± 18.1 %, respectively, in the five SI grade 0 patients; 40.6 ± 21.1 and 66.0 ± 18.1 %, respectively, in the 20 grade 1 patients; and 26.0 ± 6.9 and 30.9 ± 26.6 %, respectively, in the 13 grade 2 patients. Preoperative sensory (touch) scores and recovery ratios (%) were 100.8 ± 7.6 and 69.8 ± 19.7 %, respectively, in the five grade 0 SI patients; 77.5 ± 29.2 and 29.7 ± 19.2 %, respectively, in the 20 grade 1 patients; and 49.4 ± 36.7 and 21.8 ± 22.2 %, respectively, in the 13 grade 2 patients. The motor and sensory recovery ratios were significantly different among the three groups (*p* < 0.05) (Table 1). That is, patients with a higher grade of intramedullary signal intensity showed poorer ASIA motor and sensory recovery ratio than those with a lower grade of signal changes on T2-weighted images (Fig. 5).

In present study, 29 patients were classified as K-line (+) and 9 were classified as K-line (−). The mean recovery ratio was 63.7 ± 24.3 % in the K-line (+) group and 30.3 ± 25.6 % in the K-line (−) group. The neurological outcome in patients with K-line (+) was better than in those with K-line (−) (*p* < 0.05).

### Logistic regression analyses

**Fig. 6:**
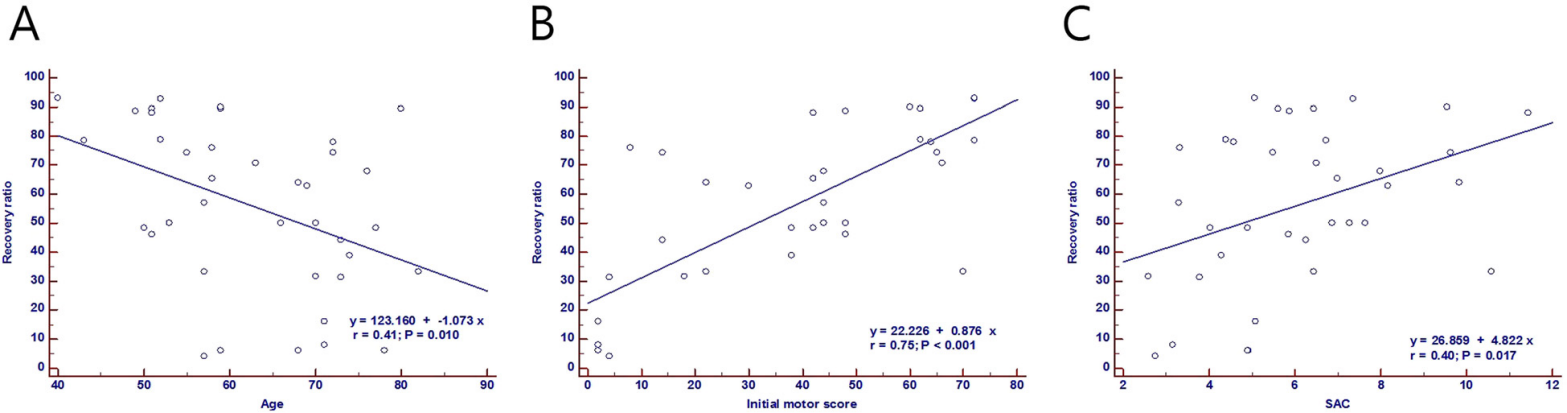
The correlation between age, ASIA motor score, SAC, and recovery ratio. **a** The patients’ age showed a significant correlation with the recovery ratio. **b** Initial ASIA motor score was correlated with recovery ratio. **c** Recovery ratio was correlated with the space available for the spinal cord (SAC)

Univariate logistic regression analysis and multivariate logistic regression analysis were performed to identify prognostic factors. Patient age, initial ASIA motor score, severity of intramedullary signal intensity, and SAC had significant effects on the neurological outcome after surgery (Table 2). Patient age was significantly correlated with recovery ratio (coefficient = −1.0733, 95 % CI = −1.8715– − 0.2752, *p* = 0.0098, Fig. 6a). Initial ASIA motor score was significantly correlated with recovery ratio (coefficient = 0.7533, 95 % CI = 0.5711–0.8647, *p* < 0.0001, Fig. 6b). Furthermore, the change in SAC was significantly correlated with the rate of postoperative neurologic improvement (coefficient = 0.3967, 95 % CI = 0.07842–0.6416, *p* = 0.0166, Fig. 6c). However, there was no significant relationship between initial cervical curvature, time interval between injury and operation, OPLL type, the length of signal intensity (SI), or congenital canal stenosis (Pavlov’s ratio) and neurological outcome.

**Table 2:** Multivariate regression analysis results for the recovery ratio and other factors

Recovery ratio	Coef.	s.e.	*t* value	*p* value	95 % CI
Age	−1.073	0.394	−2.727	0.0098	−1.872 to -0.275
Operation interval	0.737	1.151	0.640	0.526 NS	−1.598 to 3.071
Cervical alignment	0.284	0.611	0.464	0.645 NS	−0.956 to 1.523
Cervical stenosis (Pavlov ratio)	1.958	25.642	0.0763	0.939 NS	−50.099 to 54.014
SAC	4.822	1.913	2.520	0.016	0.934 to 8.711
SI length	−0.868	0.660	−1.316	0.196 NS	−2.207 to 0.470
SI grade	−7.996	5.328	−5.254	<0.001	−8.802 to -3.189
Preoperative motor score	0.876	0.128	6.872	<0.001	0.618 to 1.135

*SAC* space available for the spinal cord, *SI* signal intensity, *Coef.* regression coefficient, *s.e.* standard error, *NS* non-significant

In our study, 1 of the 38 patients (2.6 %) developed C5 palsy and postoperative hematoma 3 h after surgery. We immediately removed the hematoma, but the C5 nerve palsy remained on the patient’s right side. Another patient developed superficial wound infection after surgery, but no additional surgery was required to remove instrumentation.

## Discussion

OPLL has been found to be highly prevalent in patients with acute traumatic cervical SCI; in particular, OPLL has been reported in 34 ~ 38 % of cervical SCI patients without bone injury [1, 2]. They were caused primarily by low-energy trauma. The vast majority of patients were elderly and unaware of OPLL. According to other studies [16], there are only a few patients who are aware of having cervical OPLL before trauma. In our cases, one patient suffered neck pain 2 years before the trauma but he was not aware of cervical OPLL and was not diagnosed with cervical OPLL. Consistent with previous studies, the majority of SCI patients with OPLL in our study were elderly, with the peak incidence found in patients between 65 and 75 years old of age.

### Intramedullary signal changes and neurological outcome

Several studies have investigated the relationship between increased SI on MRI and surgical outcomes in patients with acute cervical cord injury [3, 5, 6, 17, 18]. We found that if intramedullary signal changes on T2WI were present, clinical outcomes were unfavorable after surgery as compared to those patients with no intramedullary signal changes on T2WI. We recognized three grades of cord SI and found that these grades were inversely proportional to the outcome of cervical cord injury. Patients with a brighter SI change (grade 2) on T2-weighted MR images had poorer neurological outcomes than those with a faint SI change (grade 1) or those without a SI change (grade 0). Thus, decompressive surgery should be performed for patients with high intramedullary SI if possible.

Ramanauskas and colleagues [19] divided myelomalacia into three stages and speculated that in the early stage, a change in the signal intensity of the spinal cord on MRI reflects cord edema. In contrast, in the intermediate stage, a signal change reflected cystic necrosis of the central gray matter after prolonged cord edema. They reported that in early and intermediate stages, the spinal cord showed high-signal intensity on T2WI sequences, while at a later stage, low-signal intensity on T1WI and high SI on T2WI sequences were noted. We did not find a low-signal change on T1-weighted images initially. We assumed that this was because patients experienced a sudden minor traumatic force to their neck area, and there was not sufficient time for low SI changes to develop on T1WI.

### Age and neurological outcome

Age as an adverse prognostic factor for neurological outcome has been reported previously [4, 20]. Prognosis was less optimistic in patients with severe initial cord injury who were older than those who were younger [20]. We also found that older age at injury was a negative predictor of neurological recovery. Older patients tend to have a higher incidence of cervical spondylosis and cord ischemia due to atherosclerotic changes of the vertebral or feeding vascular structures. These senile factors and other comorbidities might contribute to unfavorable clinical outcomes in elderly patients with an injured spinal cord and OPLL.

### Canal stenosis and neurological outcome

Minor trauma to the neck sometimes results in deterioration of symptoms in patients with cervical OPLL because of preexisting spinal canal narrowing by OPLL [21–23]. Previous investigators noted poor neurological outcome in spinal cord injured patients who had severe cord compression [24–26]. Furthermore, developmental cervical spinal canal stenosis may lead to transient compression of the spinal cord and affect the neurological outcome after surgery [23]. In the present study, we found that the extent of cord compression, as assessed by parameters such as SAC, was correlated with neurologic recovery in SCI patients with OPLL, but the effect of the developing cervical canal on neurological recovery was not significant. At the moment of traumatic injury, the spinal cord can be abruptly pinched by inner structures of the spinal canal, particularly in severe stenotic areas caused by OPLL. The traumatic force may cause intramedullary SI changes to develop, resulting in neurologic deficits. Lesions of intramedullary SI change were in areas adjacent to the severely compressed spinal cord. We found that the extent of cord compression was a more important predictor of neurological outcome after surgery than congenital canal stenosis (Pavlov’s ratio) in cervical cord injured patients with OPLL.

### Initial neurological status and neurological outcome

Initial ASIA motor score has been shown to be significantly related to ambulatory and hand function outcomes in patients with spinal cord injury [21, 25]. In agreement with other studies [21, 26–28], we found that preoperative neurological status, particularly motor score, predicted neurological recovery. One interesting finding of this study was that the ASIA sensory recovery ratio was lower than the ASIA motor recovery ratio. This suggests that if patients with minor traumatic injury have high intramedullary SI changes in the spinal cord, their sensory function improves less than their motor recovery.

Fujiyoshi and colleagues [14] reported the K-line as a simple and practical index for deciding the surgical approach for cervical OPLL patients and asserted the correlation of cervical alignment with neurological outcome. They advocated that the patients with a negative K-line slope, which meant the OPLL touched the spinal cord, had a significantly lower recovery rate than those with a positive K-line. In accordance with previous studies [14, 29, 30], our present study showed the better recovery rate in the patients with positive K-line than in negative K-line.

Limitations of this study include its retrospective design and the small number of patients. The type of trauma may also have been more varied than we accounted for because it was patient-reported. Furthermore, the definition of a minor traumatic injury is controversial. Despite these drawbacks, our results provide insight into the factors that predict neurological recovery in patients with OPLL who undergo decompression surgery due to an injured cervical cord. We did not perform a quantitative analysis of signal intensity in the spinal cord. Rather, radiologists blinded to the patient data categorized intramedullary signal intensities into three groups. Further extended and comprehensive studies are required to confirm the specific relationships between prognostic factors and clinical outcomes described in this paper.

## Conclusions

The degree of canal stenosis, including SAC, intramedullary signal intensity change, initial motor score, and patient age, should be considered prognostic factors when surgical decompression is performed for cervical cord injured patients without bony fractures, particularly for those patients who have a high intramedullary SI initially. That is, prognosis was less optimistic in older patients with severe neurologic deficits, marked cord compression, or the existence of abnormal MR signal change in the spinal cord.
